# Supplementary material for: Disease related changes in ATAC-seq of iPSC-derived motor neuron lines from ALS patients and controls
Source: Nat Commun. 2024 May 2;15:3606. doi: 10.1038/s41467-024-47758-8 (PMC11066062; doi:10.1038/s41467-024-47758-8)
Supplement: Supplementary file 1 — Supplementary Information [file 41467_2024_47758_MOESM1_ESM.pdf]

Supplementary Information

Disease-related changes in ATAC-Seq of more than 450 iPSC-derived motor neuron lines from ALS patients and controls

Stanislav Tsitkov[1], Kelsey Valentine[1], Velina Kozareva[1], Aneesh Donde[1], Aaron Frank[2], Susan Lei[2], the Answer ALS Consortium, Jennifer Van Eyk[3], Steve Finkbeiner[4,5,6], Jeffrey Rothstein[7,8], Leslie Thompson[9,10,11,12], Dhruv Sareen[2,13], Clive N. Svendsen[2,13], and Ernest Fraenkel[1,+]

[1]Department of Biological Engineering, Massachusetts Institute of Technology, Cambridge, MA, USA. [2]Cedars-Sinai Biomanufacturing Center, Cedars-Sinai Medical Center, Los Angeles, CA, USA. [3]Advanced Clinical Biosystems Research Institute, Cedars-Sinai Medical Center, Los Angeles, CA, USA. [4]Center for Systems and Therapeutics, Gladstone Institutes, University of California, San Francisco, San Francisco, CA, USA. [5]Taube/Koret Center for Neurodegenerative Disease, Gladstone Institutes, University of California, San Francisco, San Francisco, CA, USA. [6]Departments of Neurology and Physiology, University of California, San Francisco, San Francisco, CA, USA. [7]Brain Science Institute, Johns Hopkins University School of Medicine, Baltimore, MD, USA. [8]Department of Neurology, Johns Hopkins University School of Medicine, Baltimore, MD, USA. [9]Institute for Memory Impairments and Neurological Disorders, University of California, Irvine, CA, USA. [10]Department of Biological Chemistry, University of California, Irvine, CA, USA. [11]Department of Neurobiology and Behavior, University of California, Irvine, CA, USA. [12]Department of Psychiatry and Human Behavior and Sue and Bill Gross Stem Cell Center, University of California, Irvine, CA, USA. [13]The Board of Governors Regenerative Medicine Institute, Cedars-Sinai Medical Center, Los Angeles, CA, USA.

[+]Corresponding Author

Contact:

Ernest Fraenkel
fraenkel-admin@mit.edu

Table of Contents

Supplementary Information Section 1: **Batch control design** 3

Supplementary Information Section 2: **Reproducibility analysis** 3

Supplementary Information Figure S1: **Alignment quality control** 4

Supplementary Information Figure S2: **Replicate analysis** 5

Supplementary Information Section 3: **PCA analysis** 6

Supplementary Information Section 4: **Example of genomic variant in B(D/T)C sample** 6

Supplementary Information Figure S3: **Drivers of variation in most variably accessible regions** 7

Supplementary Information Section 5: **Motif Enrichment Analysis of 1000 most variably accessible regions** 9

Supplementary Information Figure S4**. Drivers of variation in most variably accessible regions.** 9

Supplementary Information Section 6: **Sequencing-associated covariates** 10

Supplementary Information Figure S5: **Influence of sequencer on chromatin accessibility** 11

Supplementary Information Figure S6: **Chromatin accessibility of regions associated with ALS genes** 13

Supplementary Information Section 7: **Correlations between chromatin accessibility and the S100B staining marker** 14

Supplementary Information Section 8: **Confirmation of X-chromosome inactivation** 15

Supplementary Information Figure S7: **Clinical and demographic covariates extended data** 16

Supplementary Information Section 9: **ALS-associated differential analyses** 17

Supplementary Information Figure S8: **AALS ATAC-seq ALS signals** 19

Supplementary Table Captions 20

References 22

Supplementary Information Section 1
Batch control design

The scale of the study required that patient samples be batched for both differentiation and sequencing. A maximum of 12 lines were differentiated in each batch. These were collected into larger batches of 22-36 lines for sequencing. Accordingly, several quality control (QC) measures were incorporated into the study design. Variation in differentiated cell type composition was evaluated by staining cell cultures with six immunocytochemical staining (ICC) markers: S100B, ISL1, NKX6-1, TUJ1, SMI32, and Nestin. Variation between differentiation batches was controlled for by re-differentiating the same iPSC line with each differentiation batch (the batch differentiation control, or BDC). Variation between sequencing batches was controlled for by resequencing the same sample with each sequencing batch (the batch technical control, or BTC) (Figure 1b). Both BDCs and BTCs were differentiated from the same female healthy control iPSC clone.

Supplementary Information Section 2
Reproducibility analysis

Differentiation batch and sequencing batch variation in ATAC-seq quality as measured by sequencing QC metrics, annotation distributions, and chromatin accessibility of motor neuron-specific genes was monitored for the BDC and BTC samples (Figure S2a-c). While replicate guidelines have not been defined for bulk ATAC-seq, almost all samples fell within the ENCODE guidelines for bulk RNA-seq replicates. Most isogenic replicates (BDCs) had a Pearson correlation greater than 0.9, and most anisogenic replicates (inter-individual) had a Pearson correlation greater than 0.8 (Figure S2d). The removal of a limited number of outlying samples (3 out of 55 BDCs, 2 out of 18 BTCs, and 5 out of 493 inter-individual) improved these metrics considerably (Figure S2d-f). As a final quality control measure, 22 iPSC clones were re-differentiated, sequenced, and compared to samples in the initial cohort. Notably, 21 out of 22 replicates from the same iPSC clones clustered together when correlating samples by the 500 most variably accessible autosomal chromatin regions (MVARs) (Figure S2g). Bringing the sample correlation results together, we concluded that the data was highly reproducible.

Supplementary Figure S1

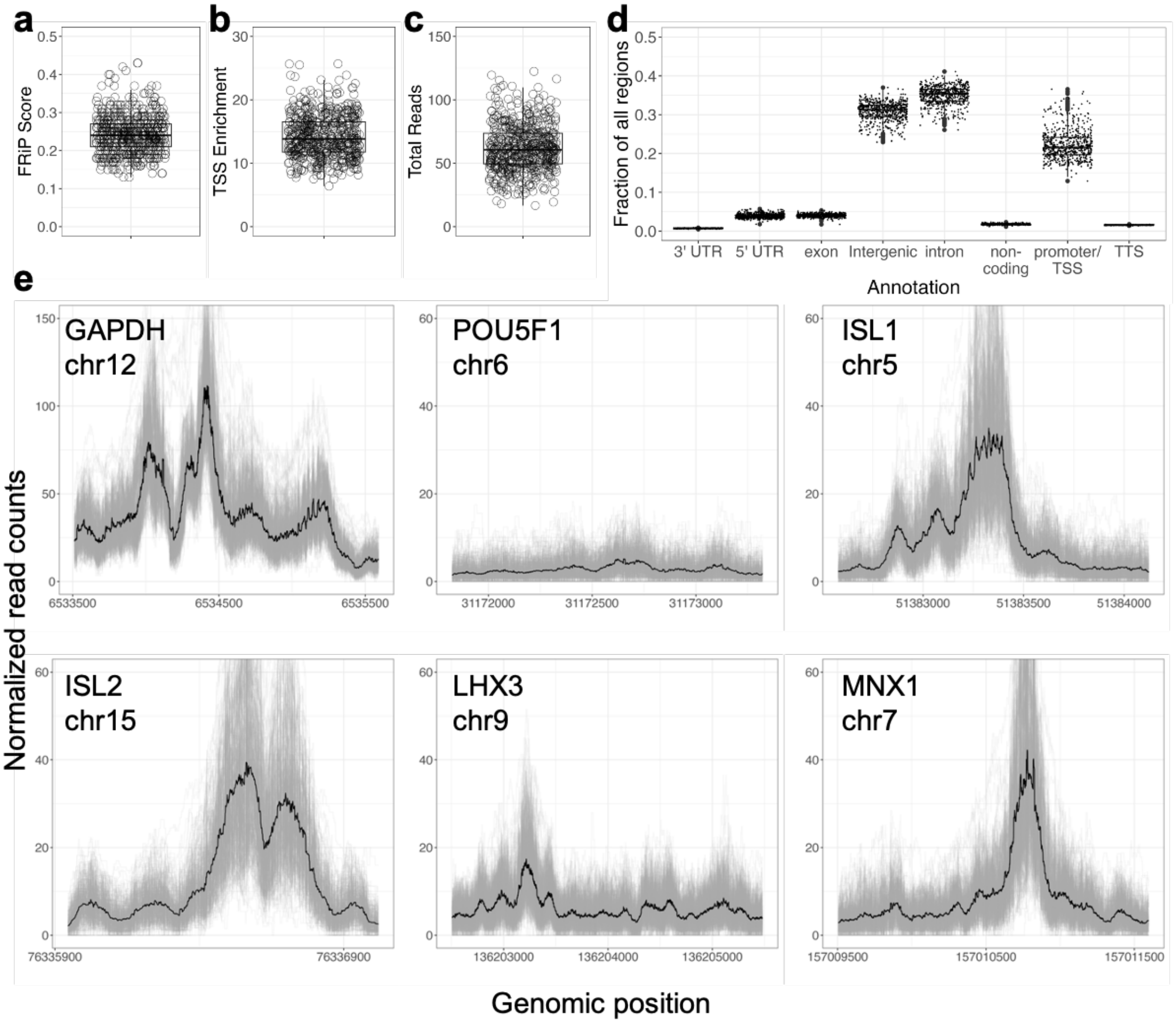

Supplementary Figure S1. Alignment quality control. a-c) Distributions of alignment QC metrics across all samples. Total reads refers to total reads (in millions) after mitochondrial filtering and deduplication. **d)** Sample-wise distributions of annotations from raw peak files. **e)** Plots of base-wise normalized coverage for all samples (gray, transparent) and the base-wise mean across all samples (black) for the chromatin regions examined in Figure 1d.

Supplementary Figure S2

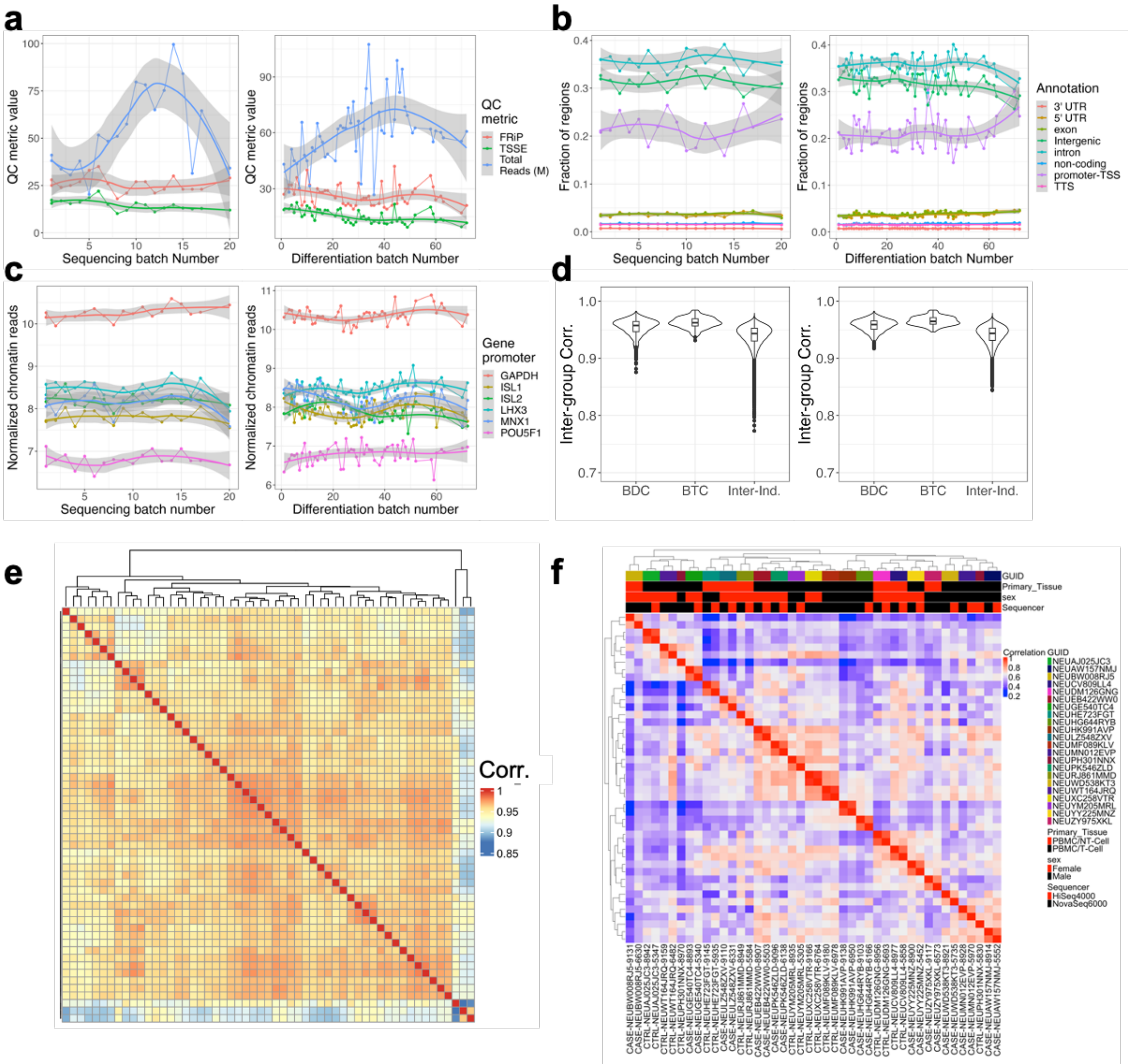

Supplementary Figure S2. Replicate analysis. a-c) Differentiation batch and sequencing batch variability in QC metrics, annotations, and motor neuron-specific chromatin regions, as measured by BDCs and BTCs, respectively. d**)** (Left) Distributions of Pearson correlation coefficients between BDC samples, between BTC samples, and between all non-B(T/D)C samples. (Right) Same as (left) but with outlying samples as identified by hierarchical clustering in (f) removed. **e)** Heatmap of correlation coefficients between BDC samples shows three outliers (last three columns). **f)** Heatmap of correlation coefficients between differentiation replicates for 500 most variably accessible regions; 21 out of 22 samples cluster together when correlating across top 500 most variably accessible regions. Regions on the sex chromosomes are not included in the analysis.

Supplementary Information Section 3

PCA analysis

In a PCA of the 500 most variably accessible regions, sex is the primary source of variance, separating samples along PC1 (Figure S3b); this separation is driven, as expected, by regions on the Y chromosome. If the sex chromosomes are excluded from the analysis, samples separate by PBMC type (Figure S3c), i.e. whether the iPSC clone of the corresponding sample was derived from a T-cell or a non-T-cell (monocyte, non-T-cell). If samples are further stratified by PBMC type, there is separation along PC1/2 that corresponds to the sequencing instrument used (HiSeq4000 or NovaSeq6000) (Figure S3d). Regressing out the effects of sample sex, PBMC type, and sequencer by fitting a linear model and running PCA on the residuals removes the separations (Figures S3e-g).

Supplementary Information Section 4:

Example of genomic variant in B(D/T)C sample

In a PCA of the 500 most variably accessible regions across all samples, we noted that the BTC/BDC samples separated from the remainder of the female samples (Figure 2a). This separation is partially driven by genomic variants specific to the BTC/BDC samples. For example, BTC/BDC samples differ from healthy controls in an intergenic differentially accessible chromatin region (DAR) on chromosome 17 (adjusted p-value <1e-100) (Figure S3h). Differential accessibility for this DAR was confirmed by examining raw read coverage (Figure S3i). Genomic analysis revealed that the BTC/BDC iPSC clone has a rare genomic structural variant, which is characterized by a 2kb deletion at this DAR, that was previously catalogued by Abel et al. in their characterization of 17,795 human genomes (locations of catalogued deletions are shown in red track in Figure S3i).[1]

Supplementary Figure S3

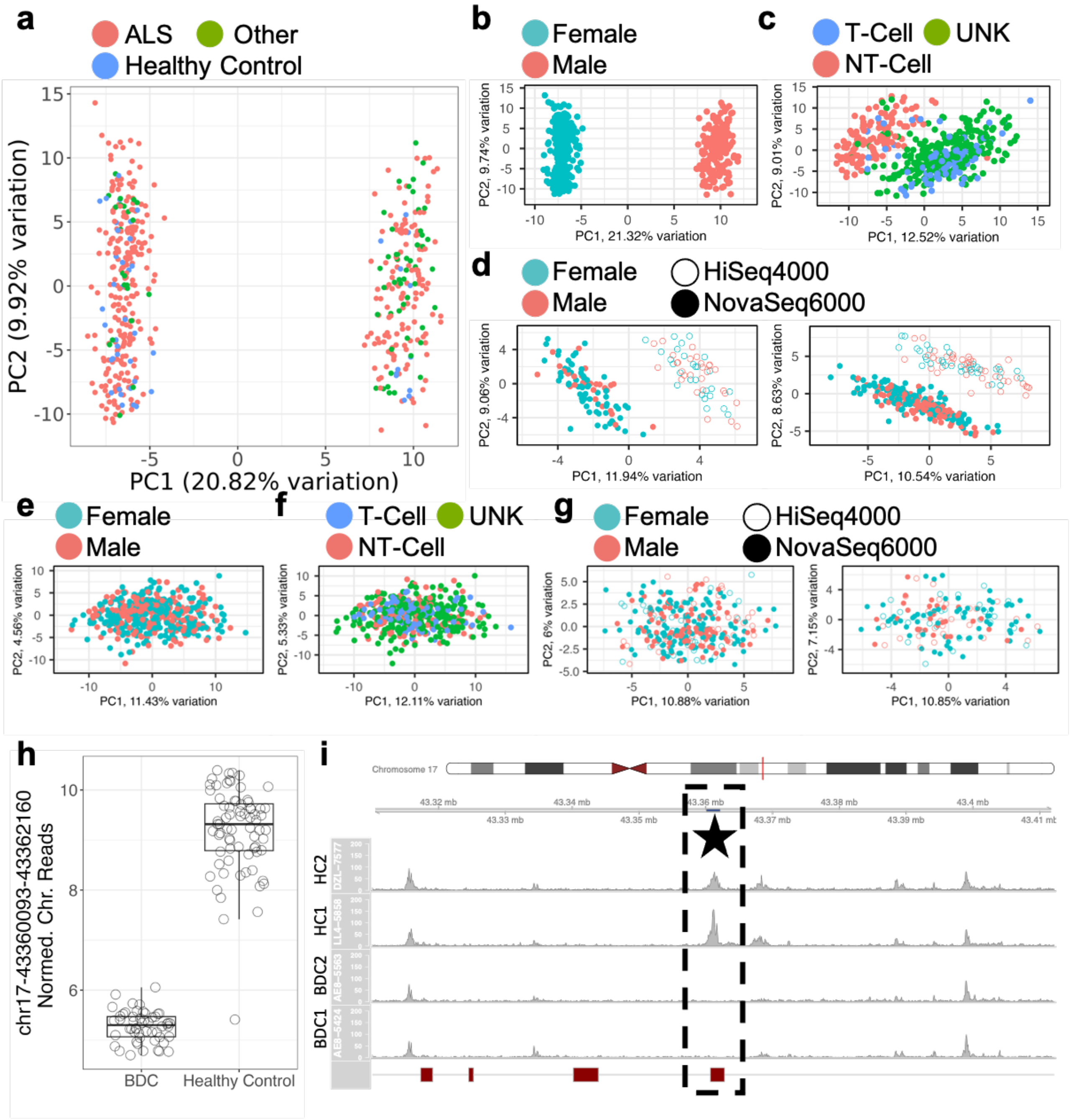

Supplementary Figure S3. Drivers of variation in most variably accessible regions. a) Biplot of PC1 and PC2 when PCA is conducted on the 500 most variably accessible regions after removing B(T/D)C samples. **b)** Same as (a) but with points labeled by sex. **c)** PC1/PC2 biplot of PCA conducted on the 500 most variably accessible regions after removing sex chromosomes. **d)** PC1/PC2 biplot of PCA conducted on the 100 most variably accessible regions after removing sex chromosomes and stratifying by PBMC type. Left, non-T-cell, right,

T-cell. **e-g)** PC1/PC2 biplots of PCA conducted on the same sets of regions as (b-d), respectively, but after FRiP score, sex, PBMC type, and sequencer have been regressed out using a linear model. **h**) BDC samples exhibit a significantly differentially accessible region (DAR) at an intergenic position on chromosome 17 (adj. p-value < 1e-100). **i)** Raw read coverage plots of the DAR from (h) for 2 BDC samples and two healthy controls (HC). Genomic position of DAR is highlighted in dark blue in the genome axis scale (above the star) and surrounded by the dashed box. Red track at the bottom corresponds to structural genomic variants identified by Abel et al.[1]

Supplementary Information Section 5

Motif Enrichment Analysis of top 1000 most variably accessible regions

We conducted motif enrichment analysis (see Methods) of the top 1000 most variably accessible chromatin regions after excluding regions on the sex chromosomes. We found enrichment for several neuronal-associated transcription factors including LHX3 and NKX6.1 (Figure S4).

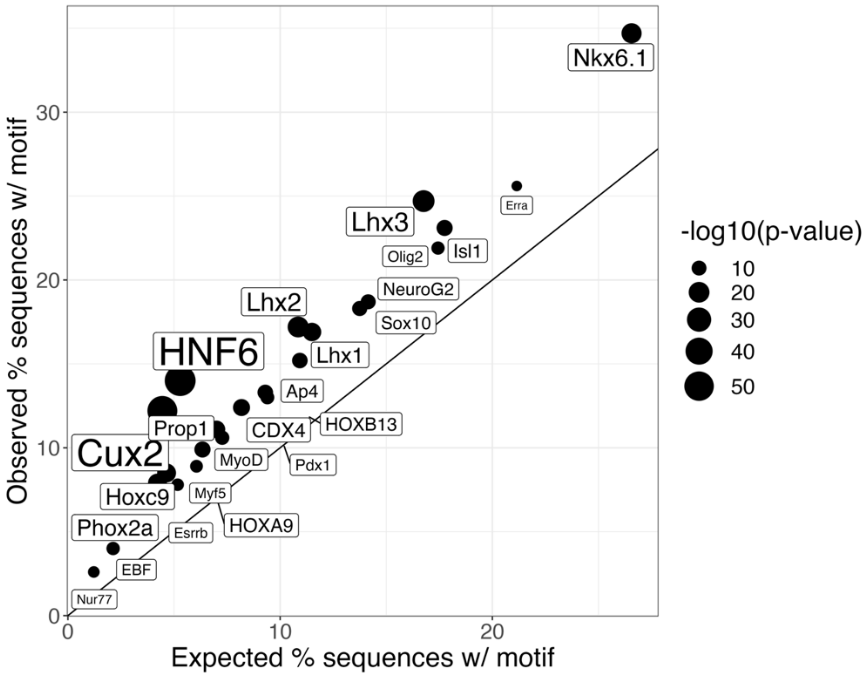

Supplementary Figure S4. Drivers of variation in most variably accessible regions. Motif enrichment analysis results for top 1000 most variably accessible chromatin regions.

Supplementary Information Section 6

Sequencing associated covariates

Several regions were highly associated with the Fraction of Reads in Peaks (FRiP) score, an ATAC-seq QC metric that reflects the signal to noise ratio of ATAC-seq data; correlations between region accessibility and FRiP score were found to be as high as 0.9 (METTL8 promoter/TSS), and as low as -0.7 (KMT2C intron). This suggested that the chosen normalization strategy using reads in peaks (RiP) did not completely remove sequencing depth associated signals (see Discussion) and led us to include FRiP score as a covariate in downstream differential analyses.

Change in sequencer from HiSeq4000 to NovaSeq6000 affected chromatin accessibility
As the result of the modernization of the sequencing facility, the instrument used for sequencing the ATAC-seq libraries changed from the HiSeq4000 to the NovaSeq6000 after the first 20 of 50 total differentiation batches (160 samples). This also resulted in a shortening of the raw sequencing read length from 75 bp to 50 bp. Comparing samples from the different platforms revealed 131 regions that differed significantly (adjusted p-value < 0.1, abs(log2FC)>1) (Figure S5a), with 118 being more accessible on the HiSeq4000 instrument (example in Figure S5b). However, there were also regions that were much more accessible when sequenced on the NovaSeq6000 (example in Figure S5c). We carried out simulations to determine the effect of read length on differential accessibility (see Methods, Figures S5d-e). Measured accessibility of the most significant DARs fell with decreasing read length (Figure S5d-f); this effect is consistent with the expectation for a low sequence complexity region. Surprisingly, one chromatin region that seemed to have significantly *lower* measured accessibility for the 75 bp HiSeq4000 samples became more accessible when reads were trimmed (Figure S5g). The raw reads that mapped to this region originally mapped to the mitochondrial genome when 75 bp long, but were mis-aligned to (non-mitochondrial) chromosome 1 when 50 bp long. A pileup of the raw reads that map to this region reveals a large number of consistent base-pair mismatches which are not observed in the genomics data for this region (Figure S5h). Based on these results, we conclude that the most significant sequencer-associated DARs are raw read length-dependent mapping artifacts. Nevertheless, while trimming reads reduced the log2FC of the most differentially accessible regions, it did not have a significant impact on overall differential analysis results. There was a similar number of DARs with a low effect size (abs(log2FC)<1) (Figs. S5d-e); both the p-value and log2FC remained similar for most genes

(Figs. S5i-j) and were largely accounted for by our corrections for FRiP and TSSE (Figure S5k-l). Since read trimming was only confirmed to eliminate a small number of DARs, we used the untrimmed data in our subsequent analyses.

Supplementary Figure S5

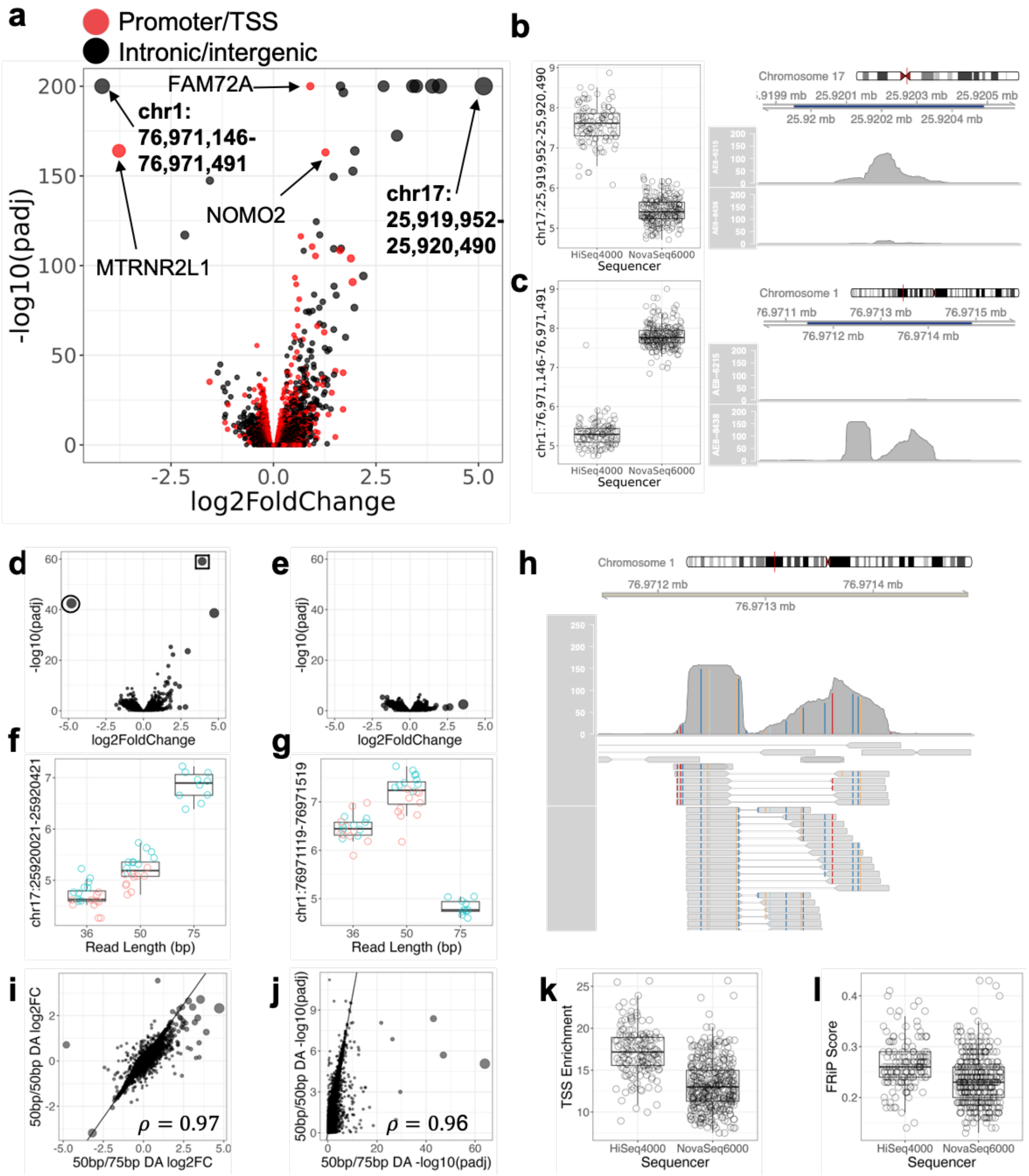

Supplementary Figure S5. Influence of sequencer on chromatin accessibility. a) Volcano plot of sequencer-associated differential signal. Point sizes reflect higher and more significant effect sizes. **b)** Normalized chromatin read counts (left) and coverage plot (right) for a BTC sample that was sequenced on the HiSeq4000 (top), and NovaSeq6000 (bottom) for the most differentially accessible region with a positive log2FC in (a) (adj. p-value <1e-278). **c)** Same as (b) but plotting the most differentially accessible region with a negative log2FC in (a) (adj. p-value<1e-278). **d)** Volcano plot for sequencer-associated differential signal for test case of 10 HiSeq4000 75 bp read samples against 10 NovaSeq6000 50 bp read samples. There are 146 DARs (p.adj<0.1), of which 75 have a large effect size (abs(log2FC)>1) and 71 have a small effect size (abs(log2FC)<1). **e)** Volcano plot for sequencer-associated differential signal from the same samples from (d), but the 75 bp HiSeq4000 reads trimmed down to 50 bp reads. There are 97 DARs (p.adj<0.1), of which 3 have a large effect size (abs(log2FC)>1) and 94 have a small effect size (abs(log2FC)<1). **f)** Plot of normalized chromatin reads as a function of trim length for region highlighted with a box in (d). **g)** Plot of normalized chromatin reads as a function of trim length for region highlighted with a circle in (d). **h)** Coverage and pileup plots of DAR from (e) when trimmed to 50 bp. Colored lines identify mismatches to reference genome: red – G, orange – C, dark blue – T, light blue – A. **i-j)** Comparison of differential accessibility signal between original 75bp/50bp analysis and trimmed 50bp/50bp analysis, with plots of log2FC (g) and -log10(padj) (h). Black line is a reference line with a slope of 1 and intercept of 0. Pearson correlations are given in bottom right corners. **k)** Boxplot comparing the transcription start site enrichment of samples that were sequenced on the HiSeq4000 and NovaSeq6000 (Student t-test p value<2e-16). **l)** Boxplot comparing the FRiP score of samples that were sequenced on the HiSeq4000 and NovaSeq6000 (Student t-test p value = 1e-11).

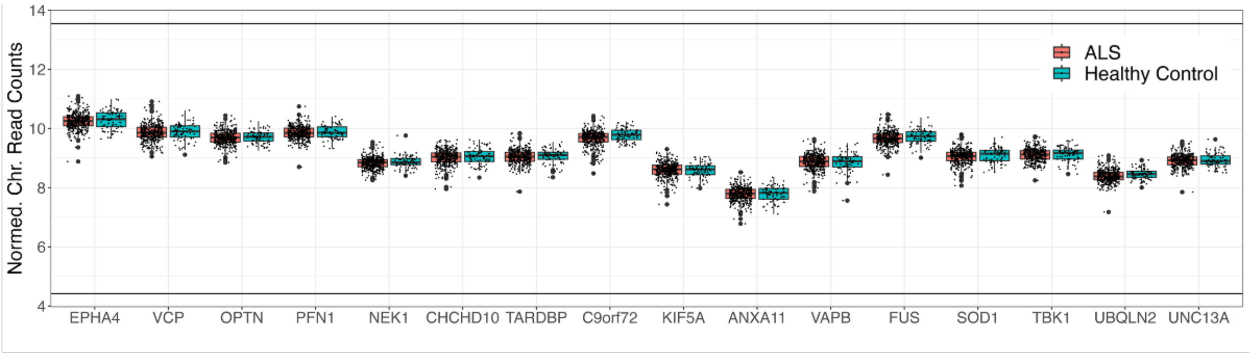

Supplementary Figure S6. Chromatin accessibility of regions associated with ALS genes. Selected chromatin regions correspond to genes that are labeled as "Definitive ALS gene" or "Clinical modifier" in the ALSoD.[2]

Supplementary Information Section 7:

Correlations between chromatin accessibility and the S100B staining marker

The associations between chromatin accessibility and ICC staining markers were notably weaker than those in gene expression data. Nevertheless, we did find that the percent of cells that stained positive for S100B (percent S100B) significantly correlated with the chromatin accessibility of several regions. Percent S100B was most highly correlated with the accessibility of an intron of *VPS13D* (ρ=0.67). The promoter region most correlated to percent S100B corresponded to that of *WBP1L* (ρ=0.66). Interestingly, the gene expression of *WBP1L* has a correlation of only 0.50 with percent S100B. A similar effect was observed in the RNA-seq data, where the expression of the *S100B* gene was less correlated with percent S100B than several other genes. This suggests that there is an additional level of regulation beyond gene expression and chromatin accessibility that drives the percent S100B-associated signal in this ATAC-seq data.

Supplementary Information Section 8

Confirmation of X-chromosome inactivation

A source of variability in the use of iPSC-derived cell models of disease is the erosion of X chromosome inactivation which can lead to imbalanced gene expression.[3] This is especially important if the disease has an X-linked component, and ALS has been characterized to be more prevalent in males.[4] So, our goal was to determine whether the X chromosome was inactivated. On the one hand, the chromatin region corresponding to the housekeeping gene, *G6PD*, which is on the X chromosome, exhibits the same accessibility between males and females (Figure 4d). Beyond this, the volcano plot (Figure S4c) identified a distinct set of 22 regions that likely escape inactivation, exhibiting a log2FC greater than 1; they corresponded to the promoter/TSS of *XIST* (Figure 4c), and the introns of *DANT1/2* and *FIRRE*. The gene *XIST* is a known escape gene. On the other hand, there are over 1000 regions on the X chromosome that exhibit a significantly higher measured accessibility in females but with a abs(log2FC)<1. For these regions, we hypothesized that slightly elevated signal in females was due to background reads from the inactivated X chromosome. Indeed, we found that background reads on the X chromosome were 50% higher in female samples than male samples, as measured by reads *not* in peaks normalized to total reads across all chromosomes (Figure S4e). Because this higher baseline would also result in inflated reads in peaks, we concluded that the X chromosome had indeed been inactivated, consistent with previous observations of gene expression.[5]

Supplementary Figure S7

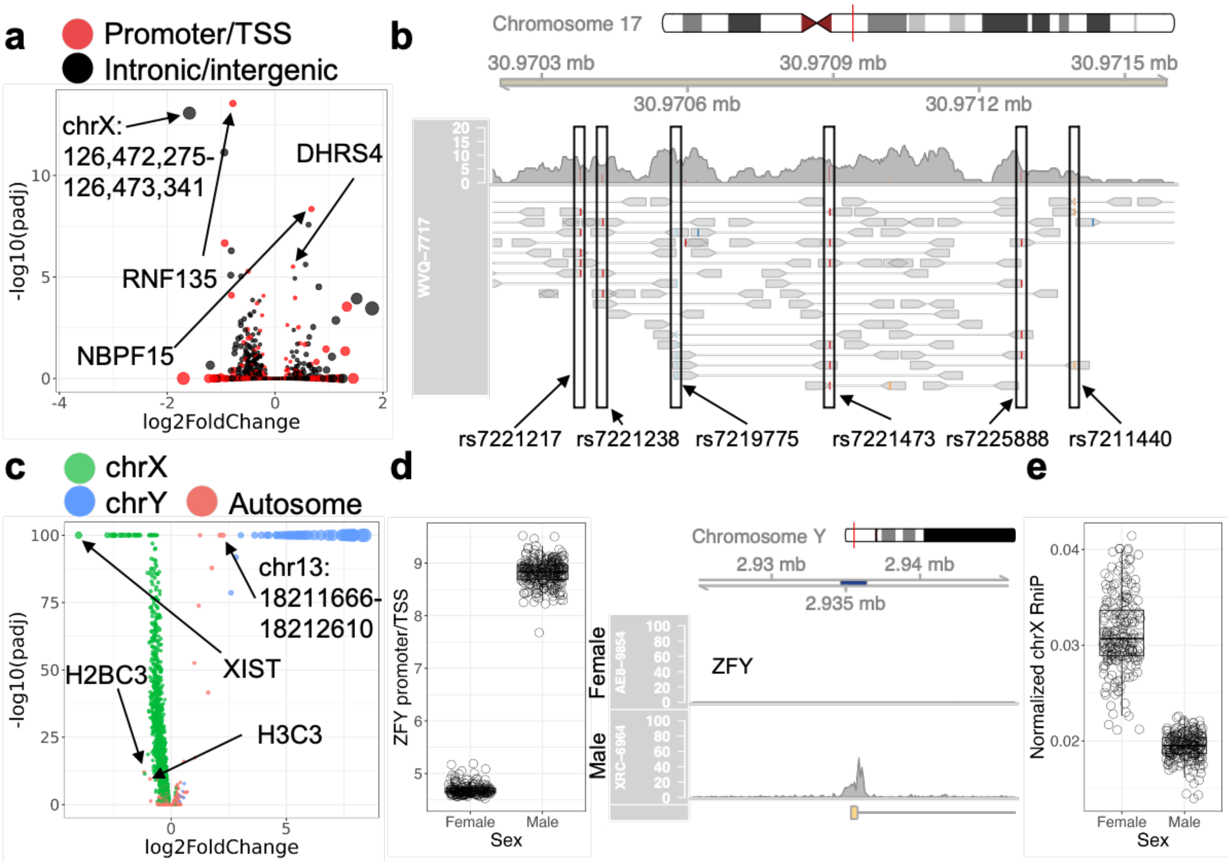

Supplementary Figure S7. Clinical and demographic covariates extended data. a) Volcano plot for ancestry-associated differential signal. **b)** Pileup and coverage plot of reads that mapped to the promoter/TSS of *RNF135*, a chromatin region whose accessibility was highly associated with ancestry for an individual of African ancestry. Boxes highlight consistent mismatches within mapped reads. **c)** Volcano plot for sex-associated differential signal. **d)** Normalized chromatin read counts (left) and example coverage plot (right) for *ZFY*, the most significant DAR on chromosome Y (adj. p-value < 1e-278). **e)** Reads **not** in peaks for chrX normalized to total reads across all chromosomes for female and male samples (p-value < 2e-16).

Supplementary Information Section 9

ALS-associated differential analyses

We conducted over two dozen differential analyses associated with ALS case status, the results of which are shown below. For each comparison, in addition to the variable being evaluated, we incorporated FRiP score, sex, PBMC type, and sequencer as covariates in the design. We found that the results of the analyses were highly dependent on the significance level and the multiple hypothesis testing correction used, and whether or not regions are filtered according to expression as described by Love et al.[6] Of the comparisons conducted, the length of disease and ALS case/healthy control comparisons had the most DARs, but only when the most lenient significance criteria were used. The most significant p-values are associated with the accessibility of the C9orf72 TSS in the C9+/C9- ALS and C9+ ALS/CTR comparisons.

Table SI-1: Number of DARs for ALS associated comparisons. B-H, 0.1: Benjamini-Hochberg adjusted p-value of 0.1 with independent filtering as described by Love et al. (B-H, 0.1). B-H, 0.1, noIndepFilt: a B-H adjusted p-value of 0.1 without independent filtering. B-H, 0.01: B-H adjusted p-value of 0.01 with independent filtering. B-H 0.01, noIndepFilt: a B-H adjusted p-value of 0.01 with independent filtering. Bonferroni, 0.01: Bonferroni adjusted p-value with independent filtering. The numbers of cases and controls in each comparison vary as a result of missing data. Some comparisons do not include controls, as they are not relevant to the analysis (for example, disease progression rates). All unadjusted p-values are calculated using a Wald test as implemented in DESeq2.

Comparison name	B-H, 0.1	B-H, 0.1 noIndepFilt	B-H, 0.01	Bonf., 0.01	Num CASE	Num CTRL	Lowest pval
Years since onset	2601	1774	28	1	337	0	1.E-07
Limb Onset	2209	0	0	0	285	75	1.E-06
Riluzole	1533	0	0	0	236	75	1.E-05
ALS vs CTR	1402	0	0	0	377	75	1.E-05
Baclofen	471	0	0	0	69	75	1.E-06
C9-/SOD1- ALS vs CTR	434	0	0	0	338	75	1.E-05
C9+ ALS vs CTR	15	15	1	1	27	75	1.E-55
Axial onset	4	4	1	1	23	75	1.E-09
Nuedexta	3	3	2	2	377	0	1.E-08
Familial ALS vs CTR	2	2	1	1	46	74	1.E-07

C9+ vs C9- ALS	1	1	1	1	137	0	1.E-56
Familial vs sporadic ALS	1	1	1	1	377	0	1.E-09
Gabapentin	1	1	1	1	31	75	1.E-07
SOD1+ vs SOD1- ALS	1	1	0	0	12	75	1.E-07
ALSFRS-R slope	0	0	0	0	213	0	1.E-05
Age of death	0	0	0	0	167	0	1.E-05
Age of onset	0	0	0	0	369	0	1.E-06
Axial onset	0	0	0	0	377	0	1.E-05
Baclofen	0	0	0	0	377	0	1.E-05
Bulbar onset	0	0	0	0	377	0	1.E-05
Gabapentin	0	0	0	0	377	0	1.E-05
Limb onset	0	0	0	0	377	0	1.E-05
Years btwn death/onset	0	0	0	0	163	0	1.E-06
Radicava	0	0	0	0	377	0	1.E-05
Riluzole	0	0	0	0	377	0	1.E-04
Bulbar onset (ALS v CTR)	0	0	0	0	99	75	1.E-04
Years until death	0	0	0	0	150	0	1.E-05
Nuedexta (ALS v CTR)	0	0	0	0	59	75	1.E-05
Radicava (ALS v CTR)	0	0	0	0	32	75	1.E-05

Supplementary Figure S8

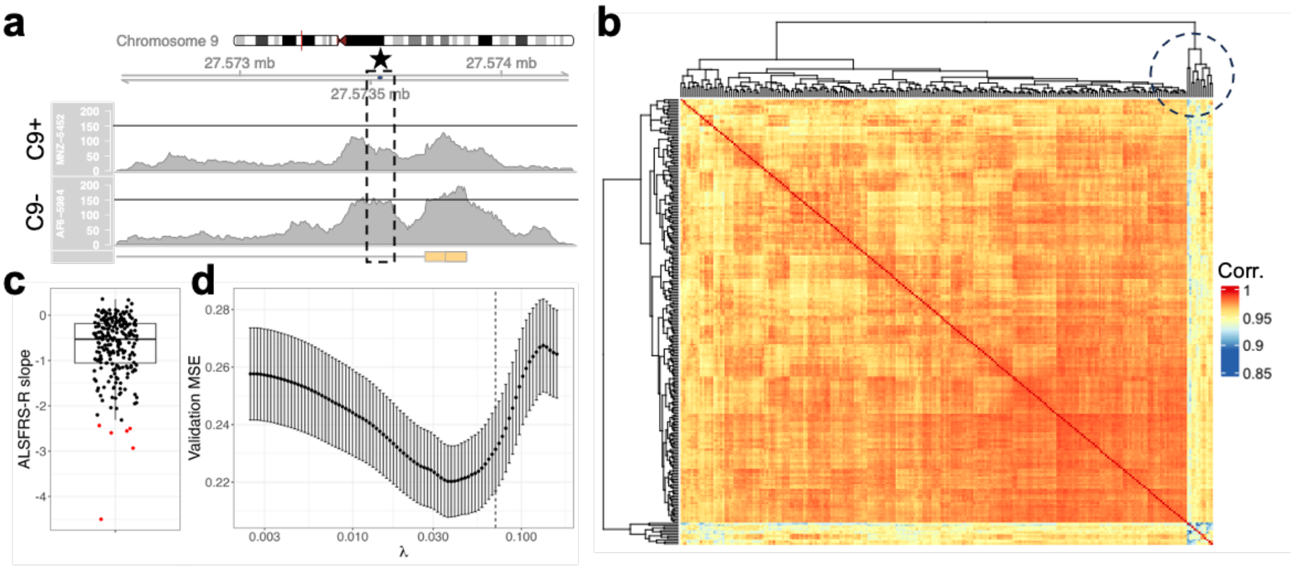

Supplementary Figure S8. AALS ATAC-Seq ALS signals. a) Example raw read coverage plot for the *C9orf72* TSS peak for a C9+ (repeat length of 274) and C9- ALS case. Region corresponding to the location of the repeat expansion is highlighted in dark blue in the genome axis scale under the star and surrounded by a box on the coverage plot. Black horizontal line is drawn at the same height in both coverage plots for reference. Note that differences in peak heights extend far beyond the region of the expansion. **b)** Correlation matrix of samples with ALSFRS-R slope using the normalized chromatin reads of regions that are within 2 kb of a TSS. Dendrogram was constructed using complete link hierarchical clustering with Euclidean distance between columns of the correlation matrix. Outlying samples are circled. **c)** Boxplot showing distribution of ALSFRS-R slopes after first round of filtering described in (a). Samples that were found to be outliers, as defined by being located more than 1.5 interquartile ranges from the first quartile, are colored in red. **d)** Example validation mean squared error (MSE) as a function of the regularization parameter, λ. Error bars denote standard error of the mean across the 10 validation folds. Dashed line corresponds to the value of λ chosen for variable selection: it is the maximum value of λ such that the validation MSE is within one standard error of the minimum validation MSE.

Supplementary Table Captions

Supplementary Table S1. Significance of association between FRiP score and chromatin accessibility as estimated by fitting a linear model with DESeq2 using FRiP score, sex, estimated PBMC type, sequencer, and ALS case status as covariates. The p-values are calculated using a two-sided Wald test as implemented in DESeq2 and adjusted using a Bonferroni correction.

Supplementary Table S2. Significance of association between sex and chromatin accessibility as estimated by fitting a linear model with DESeq2 using FRiP score, sex, estimated PBMC type, sequencer, and ALS case status as covariates. The p-values are calculated using a two-sided Wald test as implemented in DESeq2 and adjusted using a Bonferroni correction.

Supplementary Table S3. Significance of association between estimated PBMC type and chromatin accessibility as estimated by fitting a linear model with DESeq2 using FRiP score, sex, estimated PBMC type, sequencer, and ALS case status as covariates. The p-values are calculated using a two-sided Wald test as implemented in DESeq2 and adjusted for multiple hypotheses testing using a Bonferroni correction.

Supplementary Table S4. Significance of association between sequencer and chromatin accessibility as estimated by fitting a linear model with DESeq2 using FRiP score, sex, estimated PBMC type, sequencer, and ALS case status as covariates. The p-values are calculated using a two-sided Wald test as implemented in DESeq2 and adjusted for multiple hypotheses testing using a Bonferroni correction.

Supplementary Table S5. Significance of association between ALS case status and chromatin accessibility as estimated by fitting a linear model with DESeq2 using FRiP score, sex, estimated PBMC type, sequencer, and ALS case status as covariates. The p-values are calculated using a two-sided Wald test as implemented in DESeq2 and adjusted for multiple hypotheses testing using a Bonferroni correction.

Supplementary Table S6. Significance of association between years since disease onset and chromatin accessibility as estimated by fitting a linear model with DESeq2 using FRiP score, sex, estimated PBMC type, sequencer, and years since onset as covariates. The p-values are calculated using a two-sided Wald test as implemented in DESeq2 and adjusted for multiple hypotheses testing using a Bonferroni correction.

Supplementary Table S7. Set of samples used for feature selection (training) and for out of sample testing (testing) for LASSO linear regression on ALSFRS-R slope.

Supplementary Table S8. Set of chromatin regions identified from 1000 cross-validation runs on training samples from Table S7. Count column indicates number of times each chromatin region was returned in the final set of predictors.

Supplementary Table S9. All peak-gene pairs that exhibit a significant association between accessibility and expression (Bonferroni adj. p-value < 0.01), such that the peak is within 250 kb of the gene TSS. Each p-value is calculated using a two-sided Student's t test and adjusted for multiple hypotheses testing using a Bonferroni correction.

Supplementary Table S10. Peaks sorted by the number (and significance) of *both* positive and negative correlations they exhibit with gene expression. Last column corresponds to the

product of the log10 of the p-values of the most positive and most negative correlation, and is only used for sorting. Each p-value is calculated using a two-sided Student's t test and adjusted for multiple hypotheses testing using a Bonferroni correction.

References:

1. NHGRI Centers for Common Disease Genomics *et al.* Mapping and characterization of structural variation in 17,795 human genomes. *Nature* **583**, 83–89 (2020).
2. Abel, O., Powell, J. F., Andersen, P. M. & Al-Chalabi, A. ALSoD: A user-friendly online bioinformatics tool for amyotrophic lateral sclerosis genetics. *Hum. Mutat.* **33**, 1345–1351 (2012).
3. Mekhoubad, S. *et al.* Erosion of Dosage Compensation Impacts Human iPSC Disease Modeling. *Cell Stem Cell* **10**, 595–609 (2012).
4. Trojsi, F., D'Alvano, G., Bonavita, S. & Tedeschi, G. Genetics and Sex in the Pathogenesis of Amyotrophic Lateral Sclerosis (ALS): Is There a Link? *IJMS* **21**, 3647 (2020).
5. Workman, M. J. *et al.* Large-scale differentiation of iPSC-derived motor neurons from ALS and control subjects. *Neuron* **111**, 1191-1204.e5 (2023).
6. Love, M. I., Huber, W. & Anders, S. Moderated estimation of fold change and dispersion for RNA-seq data with DESeq2. *Genome Biol* **15**, 550 (2014).
