# Supplementary material for: Disease related changes in ATAC-seq of iPSC-derived motor neuron lines from ALS patients and controls
Source: Nat Commun. 2024 May 2;15:3606. doi: 10.1038/s41467-024-47758-8 (PMC11066062; doi:10.1038/s41467-024-47758-8)
Supplement: Supplementary file 3 — Description of Additional Supplementary Files [file 41467_2024_47758_MOESM3_ESM.pdf]

**Description of Additional Supplementary Files**

**Supplementary Data S1.** Significance of association between FRiP score and chromatin accessibility as estimated by fitting a linear model with DESeq2 using FRiP score, sex, estimated PBMC type, sequencer, and ALS case status as covariates. The p-values are calculated using a two-sided Wald test as implemented in DESeq2 and adjusted using a Bonferroni correction.

**Supplementary Data S2.** Significance of association between sex and chromatin accessibility as estimated by fitting a linear model with DESeq2 using FRiP score, sex, estimated PBMC type, sequencer, and ALS case status as covariates. The p-values are calculated using a two-sided Wald test as implemented in DESeq2 and adjusted using a Bonferroni correction.

**Supplementary Data S3.** Significance of association between estimated PBMC type and chromatin accessibility as estimated by fitting a linear model with DESeq2 using FRiP score, sex, estimated PBMC type, sequencer, and ALS case status as covariates. The p-values are calculated using a two-sided Wald test as implemented in DESeq2 and adjusted for multiple hypotheses testing using a Bonferroni correction.

**Supplementary Data S4.** Significance of association between sequencer and chromatin accessibility as estimated by fitting a linear model with DESeq2 using FRiP score, sex, estimated PBMC type, sequencer, and ALS case status as covariates. The p-values are calculated using a two-sided Wald test as implemented in DESeq2 and adjusted for multiple hypotheses testing using a Bonferroni correction.

**Supplementary Data S5.** Significance of association between ALS case status and chromatin accessibility as estimated by fitting a linear model with DESeq2 using FRiP score, sex, estimated PBMC type, sequencer, and ALS case status as covariates. The p-values are calculated using a two-sided Wald test as implemented in DESeq2 and adjusted for multiple hypotheses testing using a Bonferroni correction.

**Supplementary Data S6.** Significance of association between years since disease onset and chromatin accessibility as estimated by fitting a linear model with DESeq2 using FRiP score, sex, estimated PBMC type, sequencer, and years since onset as covariates. The p-values are calculated using a two-sided Wald test as implemented in DESeq2 and adjusted for multiple hypotheses testing using a Bonferroni correction.

**Supplementary Data S7.** Set of samples used for feature selection (training) and for out of sample testing (testing) for LASSO linear regression on ALSFRS-R slope.

**Supplementary Data S8.** Set of chromatin regions identified from 1000 cross-validation runs on training samples from Table S7. Count column indicates number of times each chromatin region was returned in the final set of predictors.

**Supplementary Data S9.** All peak-gene pairs that exhibit a significant association between accessibility and expression (Bonferroni adj. p-value < 0.01), such that the peak is within 250 kb of the gene TSS. Each p-value is calculated using a two-sided Student's t test and adjusted for multiple hypotheses testing using a Bonferroni correction.

**Supplementary Data S10.** Peaks sorted by the number (and significance) of *both* positive and negative correlations they exhibit with gene expression. Last column corresponds to the product of the log10 of the p-values of the most positive and most negative correlation, and is only used for sorting. Each p-value is calculated using a two-sided Student's t test and adjusted for multiple hypotheses testing using a Bonferroni correction.

**Supplementary Data S11.** List of sample IDs used for the general analysis of the paper, the replication cohort analysis, and the matched ATAC-seq/RNA-seq co-expression analysis.
