# Supplementary material for: Disease related changes in ATAC-seq of iPSC-derived motor neuron lines from ALS patients and controls
Source: Nat Commun. 2024 May 2;15:3606. doi: 10.1038/s41467-024-47758-8 (PMC11066062; doi:10.1038/s41467-024-47758-8)
Supplement: Supplementary file 6 — Reporting Summary [file 41467_2024_47758_MOESM6_ESM.pdf]

nature portfolio

Corresponding author(s): Ernest Fraenkel

Last updated by author(s): Feb 22, 2024

Reporting Summary

Nature Portfolio wishes to improve the reproducibility of the work that we publish. This form provides structure for consistency and transparency in reporting. For further information on Nature Portfolio policies, see our Editorial Policies and the Editorial Policy Checklist.

Statistics

For all statistical analyses, confirm that the following items are present in the figure legend, table legend, main text, or Methods section.

n/a	Confirmed	
☐	☒	The exact sample size (*n*) for each experimental group/condition, given as a discrete number and unit of measurement
☐	☒	A statement on whether measurements were taken from distinct samples or whether the same sample was measured repeatedly
☐	☒	The statistical test(s) used AND whether they are one- or two-sided<br>*Only common tests should be described solely by name; describe more complex techniques in the Methods section.*
☐	☒	A description of all covariates tested
☐	☒	A description of any assumptions or corrections, such as tests of normality and adjustment for multiple comparisons
☐	☒	A full description of the statistical parameters including central tendency (e.g. means) or other basic estimates (e.g. regression coefficient) AND variation (e.g. standard deviation) or associated estimates of uncertainty (e.g. confidence intervals)
☐	☒	For null hypothesis testing, the test statistic (e.g. *F*, *t*, *r*) with confidence intervals, effect sizes, degrees of freedom and *P* value noted<br>*Give P values as exact values whenever suitable.*
☒	☐	For Bayesian analysis, information on the choice of priors and Markov chain Monte Carlo settings
☒	☐	For hierarchical and complex designs, identification of the appropriate level for tests and full reporting of outcomes
☐	☒	Estimates of effect sizes (e.g. Cohen's *d*, Pearson's *r*), indicating how they were calculated

Our web collection on statistics for biologists contains articles on many of the points above.

Software and code

Policy information about availability of computer code

Data collection	No software was used for data collection
Data analysis	ENCODE ATAC-seq pipeline: https://github.com/ENCODE-DCC/atac-seq-pipeline<br>R package DiffBind (v2.10.0)<br>R package DESeq2 (v1.38.3)<br>R package VariancePartition (v1.28.9)<br>R package Gviz (v1.46.1)<br>R package umap (v0.2.10.0)<br>R package PCAtools (v2.14.0)<br>R package ggplot2 (v3.4.1)<br>R package glmnet (v4.1-6)<br>R package GenomicInteractions (v1.36)<br>R package Rsamtools (v2.18)<br>R package ComplexHeatmap (v2.18)<br>R package pheatmap (v1.0.12)<br>Python package tensorQTL (v1.0.9)<br>Python package pysam (v0.22.0)<br>Samtools v1.3<br>HOMER software for motif discovery and next-gen sequencing analysis (v4.11)

For manuscripts utilizing custom algorithms or software that are central to the research but not yet described in published literature, software must be made available to editors and reviewers. We strongly encourage code deposition in a community repository (e.g. GitHub). See the Nature Portfolio guidelines for submitting code & software for further information.

Data

Policy information about availability of data

All manuscripts must include a data availability statement. This statement should provide the following information, where applicable:

- Accession codes, unique identifiers, or web links for publicly available datasets
- A description of any restrictions on data availability
- For clinical datasets or third party data, please ensure that the statement adheres to our policy

Data used in the preparation of this article were obtained from the ANSWER ALS Data Portal (AALS-01184). For up-to-date information on the study, visit https://dataportal.answerals.org. Almost all data is available through the Answer ALS Data Portal following approval of a Data Use Agreement (DUA) form. At the time of writing, some of these sample files have not yet been released, but are currently being processed for release. All data and code used in this study will be made available by reasonable request to the lead contact following DUA approval.

Research involving human participants, their data, or biological material

Policy information about studies with human participants or human data. See also policy information about sex, gender (identity/presentation), and sexual orientation and race, ethnicity and racism.

Reporting on sex and gender	A portion of the manuscript examines differences in chromatin accessibility associated with sex. Patient sex was reported as part of the clinical metadata collected from patients at the eight neuromuscular clinics participating in the Answer ALS program.
Reporting on race, ethnicity, or other socially relevant groupings	A portion of the manuscript examines differences in chromatin accessibility associated with ancestry. In these analyses, ancestry was identified from whole genome sequencing data.
Population characteristics	*Describe the covariate-relevant population characteristics of the human research participants (e.g. age, genotypic information, past and current diagnosis and treatment categories). If you filled out the behavioural & social sciences study design questions and have nothing to add here, write "See above."*
Recruitment	Recruitment was completed before this study was initiated
Ethics oversight	The research was deemed "exempt" from human subjects regulations by the IRB at MIT.

Note that full information on the approval of the study protocol must also be provided in the manuscript.

Field-specific reporting

Please select the one below that is the best fit for your research. If you are not sure, read the appropriate sections before making your selection.

☒ Life sciences ☐ Behavioural & social sciences ☐ Ecological, evolutionary & environmental sciences

For a reference copy of the document with all sections, see nature.com/documents/nr-reporting-summary-flat.pdf

Life sciences study design

All studies must disclose on these points even when the disclosure is negative.

Sample size	533 samples from 460 unique donors. As this was not a clinical trial there was no predetermined sample size.
Data exclusions	Samples with poor ATAC-seq QC (extremely low FRiP score, extremely low Transcription start site enrichment, extremely low reads, no nucleosome free region in fragment length distributions) were excluded from the analysis. Any result-specific exclusions are detailed in the manuscript.
Replication	22 iPSC clones were redifferentiated as part of a replication cohort. Each differentiation batch contains a batch differentiation control. Each sequencing batch contains a batch technical control. Details can be found in SI Section 2. As described in the main text, all replication studies were successful.
Randomization	Differentiation batches were assembled to contain similar numbers of males, females, cases, and controls.
Blinding	The goal of this study was to identify associations between chromatin accessibility and clinical, demographic, and technical covariates. As this was not a clinical trial, no patient blinding was appropriate.

Reporting for specific materials, systems and methods

We require information from authors about some types of materials, experimental systems and methods used in many studies. Here, indicate whether each material, system or method listed is relevant to your study. If you are not sure if a list item applies to your research, read the appropriate section before selecting a response.

Materials & experimental systems

n/a	Involved in the study
☒	☐ Antibodies
☒	☐ Eukaryotic cell lines
☒	☐ Palaeontology and archaeology
☒	☐ Animals and other organisms
☒	☐ Clinical data
☒	☐ Dual use research of concern
☒	☐ Plants

Methods

n/a	Involved in the study
☒	☐ ChIP-seq
☒	☐ Flow cytometry
☒	☐ MRI-based neuroimaging
